# Human placenta-derived mesenchymal stem cells trigger repair system in TAA-injured rat model via antioxidant effect

**DOI:** 10.18632/aging.202348

**Published:** 2020-12-26

**Authors:** Jeeyoon Na, Joseph Song, Hyun Ho Kim, Jin Seok, Jae Yeon Kim, Ji Hye Jun, Gi Jin Kim

**Affiliations:** 1 Department of Biology, University of Pennsylvania, Philadelphia, PA 19104, USA; 2 Department of Chemistry and Biochemistry, University of California San Diego, La Jolla, CA 92093, USA; 3 College of Liberal Arts and Sciences, University of Illinois at Urbana-Champaign, Urbana, IL 61801, USA; 4 Department of Biomedical Science, CHA University, Seongnam, Republic of Korea

**Keywords:** placenta-derived mesenchymal stem cells, thioacetamide, antioxidants, liver, ovary

## Abstract

Oxidative stress induces damages of various cell types or tissues through a repetitive imbalance between the systemic manifestation of reactive oxygen species (ROS) and detoxification of the reactive intermediates. Thioacetamide (TAA) is well known for causing several degenerative diseases by oxidative stress. However, study of the antioxidant mechanisms of stem cells in TAA-injured rat model is insufficient. Therefore, we investigated the effect of placenta-derived mesenchymal stem cells (PD-MSCs) transplantation on liver and ovary of TAA-injured rat models to study the antioxidant effect in degenerative diseases. In TAA-injured rat model, PD-MSCs engrafted into damaged organ including liver and ovary in PD-MSCs transplanted groups (Tx) compared with non-transplanted groups (NTx) (*p<0.05). Transplanted PD-MSCs reduced inflammatory factors and upregulated oxidative stress factors in Tx compared with NTx (*p<0.05). Also, transplanted PD-MSCs enhanced antioxidants factors and organ functional restoration factors in Tx compared with NTx. These data show that PD-MSC transplantation triggers the regeneration of organ (e.g., liver and ovary) damaged by oxidative stress from TAA treatment via activating antioxidant factors. Therefore, these data suggest the therapeutic potential via antioxidant effect and help understand the therapeutic mechanism of PD-MSCs in damaged tissues such as in liver and reproductive system.

## INTRODUCTION

Reactive oxygen species (ROS) can be produced as a result of various cellular metabolism procedures. Although a certain level of ROS is necessary in cellular metabolism and signaling, imbalance between the oxidants and antioxidants causes cellular dysfunction and tissue damage [1]. Failure in regulating the level of oxidative stress leads to various consequences including degenerative diseases. Despite the active research, there is currently no definite solution to cure degenerative diseases. Studies have shown that stem cells can be used as a therapeutic purpose. For example, stem cell therapies are widely used in curing degenerative eye diseases by replacing lost cells in the eye [2]. Also, some studies used mesenchymal stem cells’ ability to regulate inflammatory responses as a therapy to cure degenerative and inflammatory diseases including organ fibrosis, Crohn’s disease and graft-versus-host disease [3]. One of the current approaches to curing degenerative diseases is by targeting oxidative stress using human stem cells [4].

Thioacetamide (TAA) is a thinosulfur widely known as hepatotoxin that triggers liver damage by generating ROS [5]. Oxidative stress in liver induced by TAA can cause a wide spectrum ranging from acute hepatitis to cirrhosis [6]. TAA is metabolized by hepatic cytochrome (CYP) 450 and generate TAA S-dioxide which is the active product that produce reactive oxygen species (ROS) factors and production of TGF-ß [7, 8]. Although most studies focus on the effect of TAA on the function of liver, the oxidative stress generated from TAA treatment can also affect several organs including ovary, leading to dysfunction in major female reproductive organ [9]. Previous study revealed that TAA also affects the ovary by destruction of the ovarian follicles.

Mesenchymal stem cells (MSCs) can be isolated from diverse sources such as bone marrow, adipose, umbilical cord and placenta. MSCs are known for their therapeutic effects because of their self-renewal ability and multipotency. Placenta derived mesenchymal stem cells (PD-MSCs) are one of the multipotent stem cells separated from the human placenta which is expulsed during childbirth. PD-MSCs are favorably used in research since the yield of stem cells is higher in placenta compared to other stem cell sources. There are findings mentioning the therapeutic effect of PD-MSCs on angiogenesis, osteogenesis, neurogenesis and prevention of scar formation, but there is no evidence that it regulates cellular oxidative stress [10, 11].

Therefore, the objectives of the present study are to analyze the expression of oxidative stress-related markers in liver and ovary tissues of TAA-injured rat model and their expression changes in both tissues in TAA-injured rat model by PD-MSCs transplantation. Finally, we evaluated that PD-MSC transplantation can induce liver regeneration and restore ovary function in tissues damaged by TAA treatment.

## RESULTS

### Histological analysis

**Figure 1:**
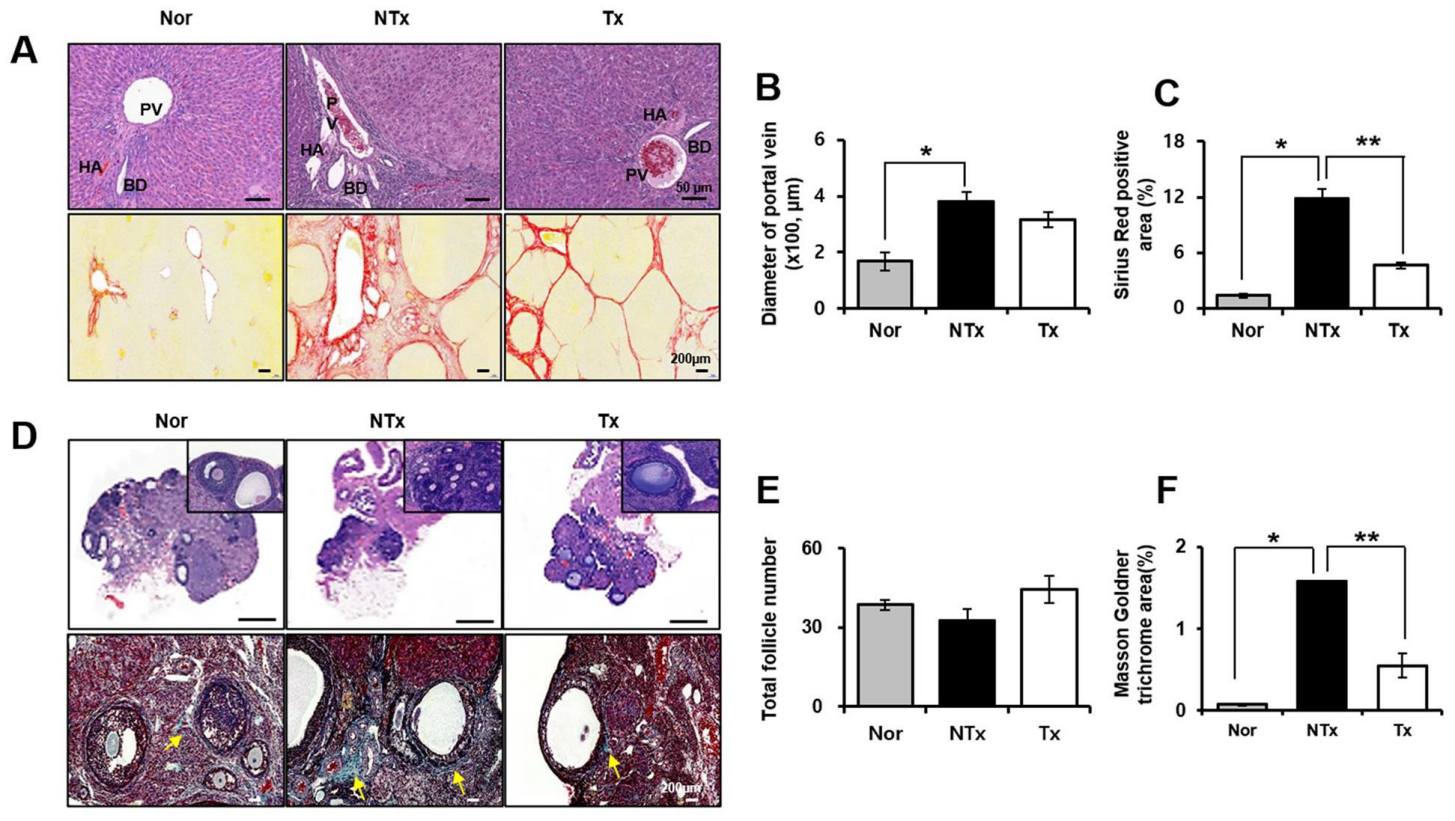
**Histological analysis in TAA-injured rat model.** H&E (**A**, upper) and Sirius red (**A**, lower) was stained and quantification (**B**, **C**) from liver in TAA-injured rat model (20x magnification). H&E (**D**, upper) and Masson Goldner trichrome (**D**, lower) was stained and quantification (**E**, **F**) from ovary in TAA-injured rat model (x20 magnification). Data represent the mean ± S.D. * Significantly different versus Normal (*p<0.05). ** Significantly different versus NTx (**p<0.05). PV, portal vein; HA, hepatic artery; BD, bile duct; SF, secondary follicle; AF, antral follicle; O, oocyte; ATF, atretic follicle.

H&E stanning results showed different tissue histology of liver and ovary in TAA-injured rat model. Histopathology of liver and ovary after PD-MSCs transplantation in TAA-injured rat was studied by conducting H&E and Sirius red staining. In non-transplantation (NTx) group, TAA-injured rat liver showed abnormal portal vein (PV), hepatic artery (HA), and bile duct (BD) as well as increase of PV diameter compared with normal liver in H&E staining as shown in Figure 1A, 1B. In addition, vascularized fibrous septa that linked portal tract with central vein were observed in NTx group by Sirius red staining * *p*<0.05, Figure 1A, 1C). Quantification of collagen deposition in NTx group was significantly increased compared with normal liver. Interestingly, liver from rats with PD-MSCs transplantation (Tx group) showed a recovery in vascular structure according to H&E staining results, which reduced extensive deposition of fibrillar collagen in Sirius red staining (* and ** *p*<0.05, Figure 1A–1C). In ovary of TAA-injured rat model, the numbers of total follicles including primordial, primary and secondary follicles were decreased in NTx group compared to the normal ovary and increased in PD-MSC Tx group compared to NTx group (Figure 1D, 1E). The follicular atresia is reported to be generated by several external factors such as oxidative stress [12]. Interestingly, follicular atresia was remarkably increased in NTx group compared to the normal ovary and decreased in PD-MSC Tx group compared to NTx group as shown Figure 1D. Furthermore, accumulation of extracellular matrix (ECM) surrounded follicles was significantly increased in NTx group compared with normal group. However, PD-MSCs transplantation mitigated the accumulation of ECM in TAA-injured rat ovary (* and ** *p*<0.05, Figure 1D, 1F). These data suggest that administration of PD-MSCs in TAA-injured rat liver results in histologically recovered structural change of liver and ovary.

### Engraftment of PD-MSCs into TAA-injured rat model

**Figure 2:**
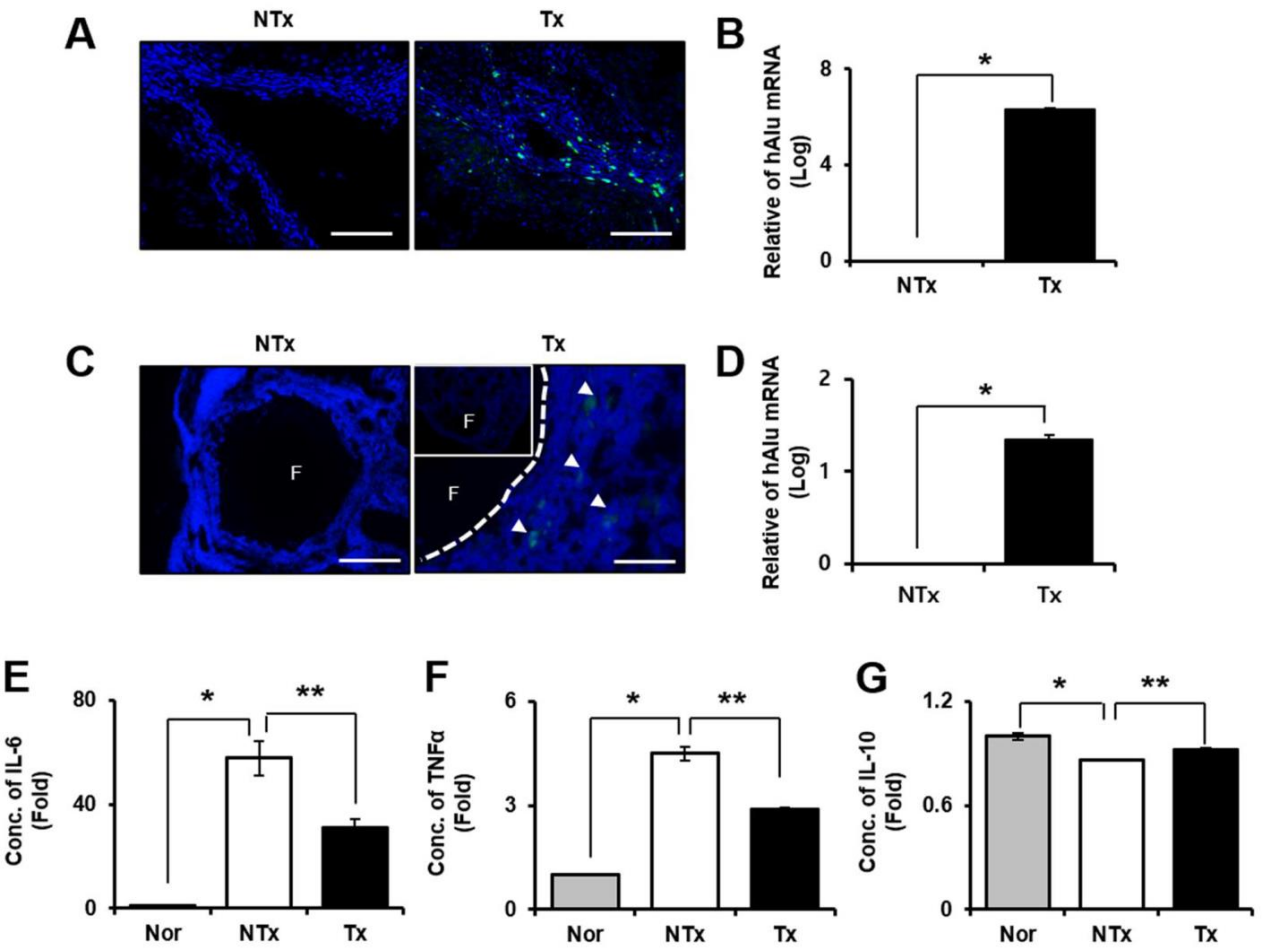
**Effect of engraftment and anti-inflammation of PD-MSCs in TAA-injured rat model.** Engraftment of PD-MSCs was determined using PKH 67 (green) labeling and human-specific alu sequence (20x magnification) from liver (**A**, **B**) and ovary (**C**, **D**) in TAA-injured rat model. Pro-inflammatory factors (**E**, **F**) and Anti-inflammatory factors (**G**) were analyzed in serum of TAA-injured rat model. Data represent the mean ± S.D. * Significantly different versus Normal (*p<0.05). ** Significantly different versus NTx (**p<0.05).

Localization of transplanted stem cells into injured target tissues is an important factor to maximize their therapeutic effects in injured site in tissues. According to the immunofluorescence data shown in Figure 2A, 2C, engraftment of PD-MSCs into target tissues including liver and ovary were traced by PKH67 fluorescence labeling dye (Figure 2A, 2C). Also, the quantification of amount of human Alu mRNA expression after PD-MSC Tx indicates the survival or the repopulation of engrafted PD-MSCs in tissues. As a result, the mRNA expressions of human Alu sequence in PD-MSC Tx group were significantly increased compared to those of NTx group in both liver and ovary * *p*<0.05, Figure 2B, 2D).

### Anti-inflammatory effect of PD-MSCs in TAA-injured rat model

We performed blood chemistry analysis for confirmation of the inflammatory response by TAA injection. The results showed that pro-inflammatory factors such as IL-6 and TNF-α were significantly decreased in Tx group compared to NTx group but increased in the NTx group compared with normal (* and ** *p*<0.05, Figure 2E, 2F). However, the anti-inflammatory factor, IL-10 was significantly increased in PD-MSC Tx group compared with NTx group (* and ** *p*<0.05, Figure 2G). These data mean that engrafted PD-MSCs to injured tissues site via transplantation reduce the inflammatory response in TAA-injured rat model.

### Antioxidant effect of PD-MSCs in TAA-injured rat liver

**Figure 3:**
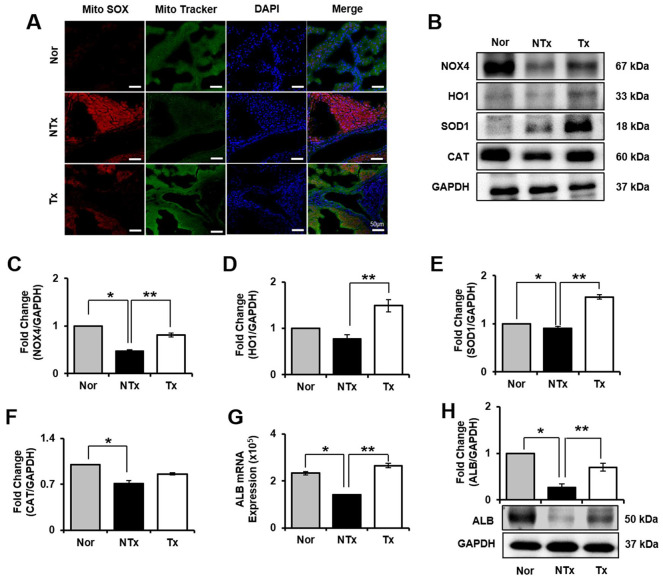
**Effect of antioxidant of PD-MSCs in liver of TAA-injured rat model.** Mito SOX and Mito Tracker were stained on liver of TAA-injured rat model by immunofluorescence (**A**). The gene expression related to antioxidants were evaluated in liver of TAA-injured rat model by western blot (**B**). Changes in protein levels are expressed (bar histogram) as band density normalized versus GAPDH (**C**–**F**). The Albumin expression of mRNA (**G**) and protein (**H**) were analyzed in liver of TAA-injured rat model by qRT-PCR and western blot. Data represent the mean ± S.D. * Significantly different versus Normal (*p<0.05). ** Significantly different versus NTx (**p<0.05).

To confirm oxidative stress in TAA-injured rat liver, we analyzed mRNA expression that encodes oxidant and antioxidant factors, such as nicotinamide adenine dinucleotide phosphatase (NADPH) oxidase 4 (NOX4), heme oxygenase-1 (HO1), superoxide dismutase 1 (SOD1), and catalase (CAT). mRNA expression of NOX4 in NTx group significantly increased compared to normal, whereas it was decreased in Tx group versus NTx (* and ** *p*<0.05, Supplementary Figure 1A). However, HO1, SOD1, and CAT was significantly decreased in NTx group compared to normal (Supplementary Figure 1B–1D). Furthermore, compared to normal liver, the expression of Mito SOX was upregulated in NTx group whereas reduced in Tx group versus NTx (Figure 3A). In addition, protein level of markers related to oxidative stress was confirmed by western blot. HO1, SOD1, CAT levels in Tx group significantly increased compared with NTx (* and ** *p*<0.05, Figure 3B, 3D–3F). These finding support that PD-MSCs have antioxidant effect that can help recover hepatic function.

### Improvement of hepatic function by PD-MSCs in TAA-injured rat model

To observe regeneration effect by PD-MSCs transplantation, we examined the protein expression of liver regeneration markers, albumin (ALB) and NOX4. NOX4 is a factor that is necessary for hepatocytes to remain its epithelial parenchymal structure [13]. We found that in both *in vivo* and *in vitro* model, liver regeneration markers, NOX4 and ALB, are significantly decreased in NTx group and increased in Tx group (* and ** *p*<0.05, Figures 3B, 3C, 3G, 3H, 5B, 5C, 5G, 5H). To determine whether the PD-MSCs improve hepatic function in TAA-injured rat model, we individually conducted serological analysis of aspartate transaminase (AST), alanine aminotransferase (ALT), total bilirubin, and albumin. Compared to normal group, the serum level of AST, ALT, and bilirubin showed significant increase in NTx group whereas the albumin level was decreased (* *p*<0.05, Supplementary Table 2). On the other hand, PD-MSCs transplantation in TAA-injured rat induced significant decrease of AST, ALT, and bilirubin levels in serum (** *p*<0.05, Supplementary Table 2). Also, albumin levels in Tx group showed remarkable improvement compared with NTx group (**p<0.05, Supplementary Table 2). These data suggest that PD-MSCs alleviate hepatic function through antioxidant process.

### Restored ovarian function by PD-MSCs through antioxidant effect

**Figure 4:**
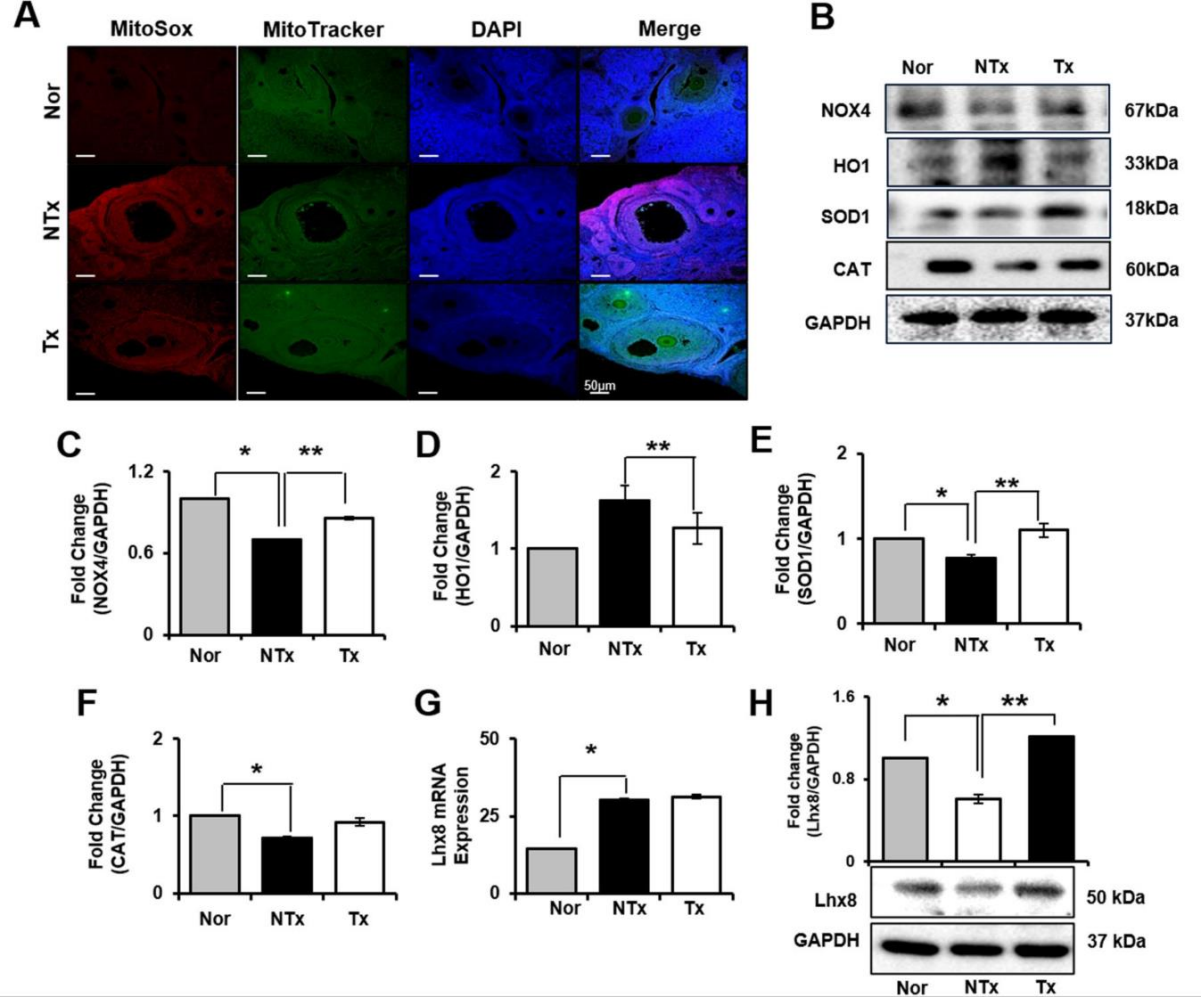
**Effect of antioxidant of PD-MSCs in ovary of TAA-injured rat model.** Mito SOX and Mito Tracker were stained on ovary of TAA-injured rat model by immunofluorescence (**A**). The gene expression related to antioxidants were evaluated in ovary of TAA-injured rat model by western blot (**B**). Changes in protein levels are expressed (bar histogram) as band density normalized versus GAPDH (**C–F**). The Lhx8 expression of mRNA (**G**) and protein (**H**) were analyzed in ovary of TAA-injured rat model by qRT-PCR and western blot. Data represent the mean ± S.D. * Significantly different versus Normal (*p<0.05). ** Significantly different versus NTx (**p<0.05).

In ovary, we also analyzed how ovary of TAA-injured rat model is affected from ROS and the roles of oxidant and antioxidant factors. As a result, mRNA expression of NOX4 and HO1 significantly increased in NTx and PD-MSC Tx group compared to normal ovary (* *p*<0.05, Supplementary Figure 2A, 2B). Based on the analysis of antioxidants factors, mRNA expression of SOD1 was significantly decreased in NTx as well as PD-MSC Tx group compared to normal ovary, and CAT was significantly increased in NTx group compared to normal and PD-MSC Tx group (* and ** *p*<0.05, Supplementary Figure 2C, 2D). Additionally, to analyze the regeneration of ovarian function, the expression of Lhx8 in ovary of TAA-injured rat model was studied. The mRNA and protein expression of Lhx8 was significantly increased in PD-MSC Tx group compared to NTx (** *p*<0.05, Figure 4H and Supplementary Figure 2E). Moreover, we analyzed antioxidant effect of PD-MSCs by IF and protein expression of related factors in ovary of TAA-injured rat model. As shown Figure 4A, Mito SOX expression in ovary, which shows ROS accumulation, was remarkably increased in NTx group compared to normal and PD-MSC Tx group (Figure 4A). The protein expression of NOX4 was decreased while the HO1 expression was significantly increased in NTx group compared to PD-MSC Tx group (* and ** *p*<0.05, Figure 4B–4D). Also, the expression of SOD1 and CAT was decreased in NTx group and significantly increased in PD-MSC Tx group (* and ** *p*<0.05, Figure 4B, 4E, 4F). These data show that PD-MSCs transplantation restored ovarian function via antioxidants effect in TAA-injured rat model.

### Antioxidant properties of PD-MSCs in primary hepatocytes treated TAA

**Figure 5:**
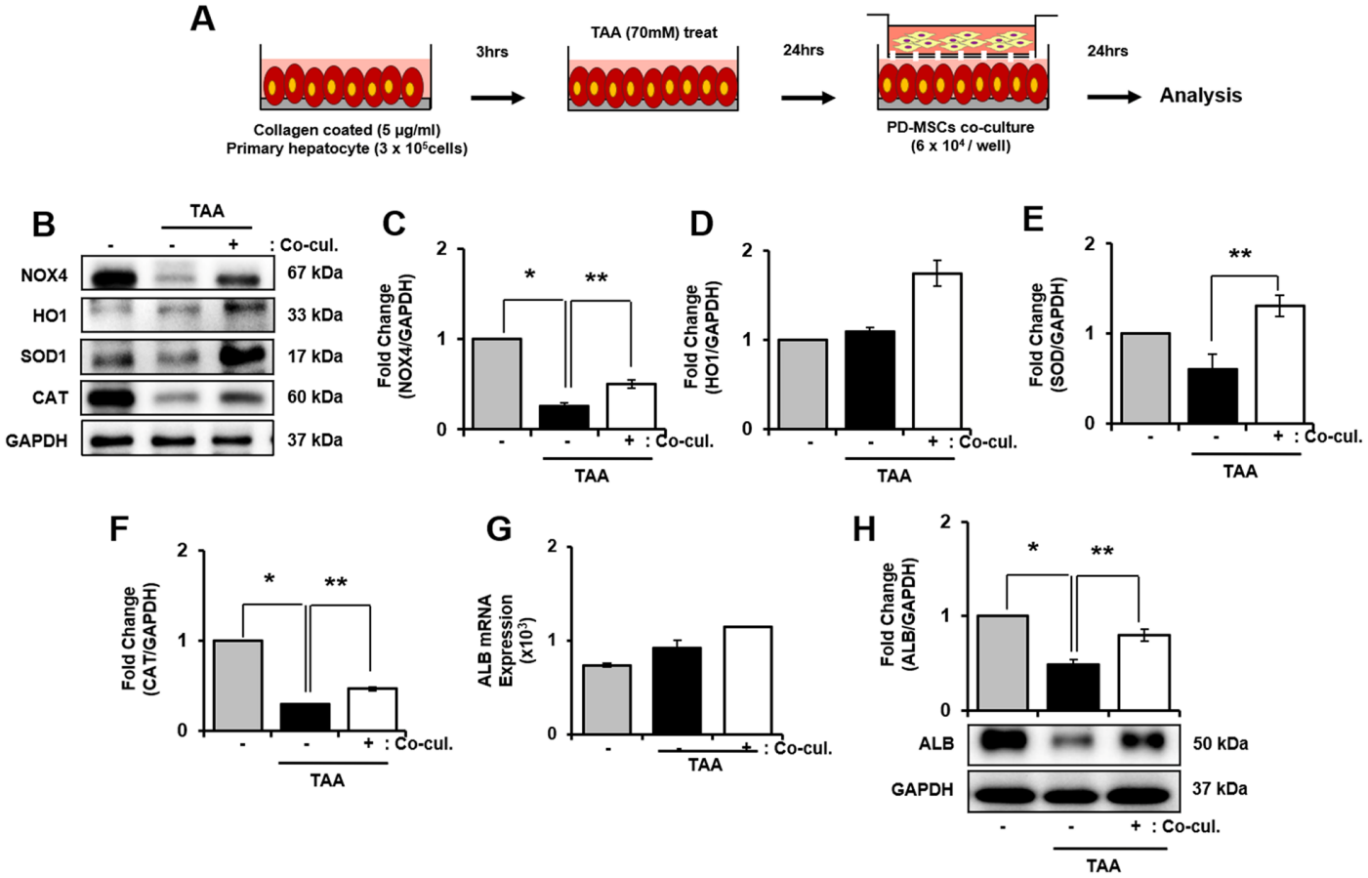
**Effect of antioxidant of PD-MSCs in TAA-treated rat primary hepatocytes.** Schematic showing the experimental protocol (**A**) The gene expression related to antioxidants were analyzed in TAA-treated rat primary hepatocytes according to PD-MSCs co-cultivation (**B**) Changes in protein levels are expressed (bar histogram) as band density normalized versus GAPDH (**C**–**F**) The Albumin expression of mRNA (**G**) and protein (**H**) were analyzed in TAA-treated rat hepatocytes according to PD-MSCs co-cultivation by qRT-PCR and western blot. Data represent the mean ± S.D. * Significantly different versus Normal (*p<0.05). ** Significantly different versus NTx (**p<0.05).

To support our finding *in vivo*, we induced oxidative stress in primary hepatocytes by treating TAA as shown Figure 5A. mRNA expression of NOX4 in TAA-injured primary hepatocytes increased, however decreased after PD-MSCs co-cultivation (Supplementary Figure 3A). In contrast, TAA treatment caused slightly decreased HO1, SOD1, and CAT in mRNA level (Supplementary Figure 3B–3D). Furthermore, HO1, SOD1, and CAT protein expression after PD-MSCs co-culture was consistently increased compared to TAA-injured primary hepatocytes (* and ** *p*<0.05, Figure 5B, 5D–5F). In addition, PD-MSCs co-cultivation significantly increased NOX4 and ALB levels (* and ** *p*<0.05, Figure 5B, 5C, 5G, 5H). These data demonstrate that co-cultivation of PD-MSCs have antioxidant effects in primary hepatocytes.

### 2.8. Antioxidant efficacy of PD-MSCs in TAA-induced ovarian explant culture

**Figure 6:**
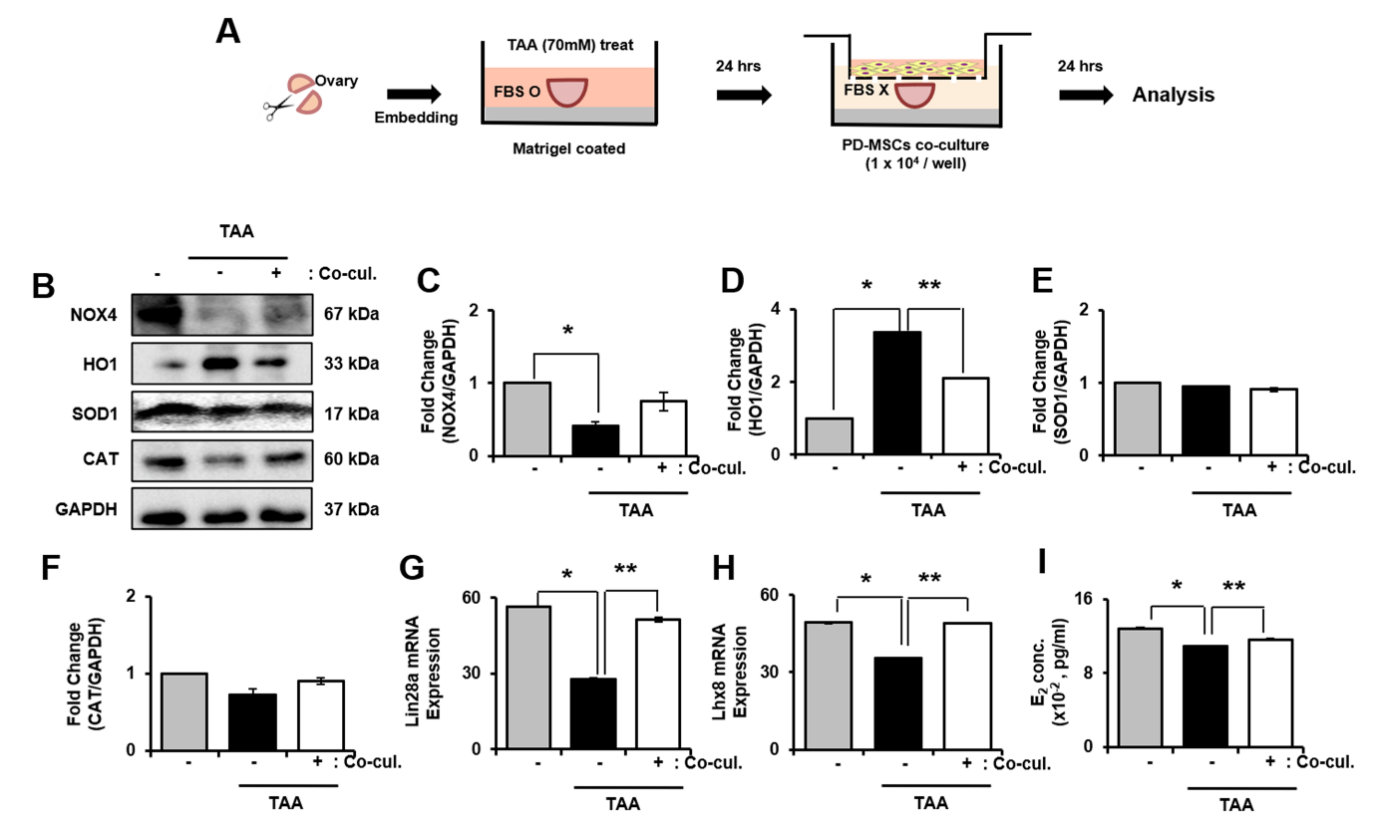
**Effect of antioxidant of PD-MSCs in TAA-treated ovary in *ex vivo*.** Schematic showing the experimental protocol (**A**) The gene expression related to antioxidants were evaluated in TAA-treated ovary according to PD-MSCs co-cultivation by western blot (**B**) Changes in protein levels are expressed (bar histogram) as band density normalized versus GAPDH (**C**–**F**) The mRNA expression of Lin28a and Lhx8 were analyzed in TAA-treated ovary according to PD-MSCs co-cultivation by qRT-PCR (**G**, **H**) The E2 hormone levels of supernatant was analyzed in supernatant of TAA-treated ovary according to PD-MSCs co-cultivation by Elisa assay (**I**) Data represent the mean ± S.D. * Significantly different Versus Normal (*p<0.05). ** Significantly different versus NTx (**p<0.05).

As explained in Figure 6A, ovary explant culture was performed. As a result, the mRNA expression of NOX4, HO1, SOD1 and CAT was significantly increased in TAA treated group compared to PD-MSCs co-cultivation group (* and ** *p*<0.05, Supplementary Figure 4A–4D). In protein level, NOX4 significantly decreased whereas expression of HO1 significantly increased in TAA treated group compared to control. In PD-MSCs co-cultivation group, expression of NOX4 increased and HO-1 decreased compared to TAA treated group (* and ** *p*<0.05, Figure 6B–6D). However, the expression of SOD1 showed no difference in TAA treated group and PD-MSCs co-cultivated group compared to control. Also, expression of CAT decreased in TAA treated group while increased in PD- MSCs co-cultivation group (Figure 6B, 6F). These data mean that PD-MSCs co-cultivation induce antioxidant effects in TAA treated ovary.

### Recovery of ovarian function by PD-MSCs co-cultivation in TAA-induced ovarian explant culture

To analyze the factors related to folliculogenesis, we analyzed expression of Lim-28 Homology A (Lin28a), LIM Homeobox 8 (Lhx8), NOBOX oogenesis homeobox (Nobox) and Nanos C2HC-Type Zinc Finger 3 (Nanos3). As a result, gene expression of Lin28a and Lhx8 were clearly decreased in TAA-induced group compared to both control and PD-MSCs co-cultivation group. Also, the gene expression of Lin28 and Lhx8 significantly increased in PD-MSCs co-cultivation group compared to TAA treated group (* and ** *p*<0.05, Figure 6G, 6H). Also, the gene expression of Nobox and Nanaos3 significantly decreased in TAA treated group compared to control while increased in PD-MSCs co-cultivation group compared to TAA-induced group (Supplementary Figure 4E, 4F). Furthermore, E2 concentration in explant culture media significantly decreased in TAA treated group compared to control and increased in PD-MSCs co-cultivation group (* and ** *p<0.05*, Figure 6I). Thus, results show that PD-MSCs co-cultivation induce folliculogenesis through antioxidant effects in TAA treated ovary.

## DISCUSSION

Stem cell therapy is becoming the new therapeutic potential in regenerative medicine. Recently, PD-MSCs are favorably used in research because they can be isolated in a non-invasive method from the placenta extracted during childbirth. Also, PD-MSCs have high yield of stem cells and able to control immune responses in various ways [14]. In the present study, we demonstrated that PD-MSCs have antioxidant effect on thioacetamide (TAA) treated rat model. Generally, oxidative stress resulting from the imbalance between oxidative and antioxidant factors is one of the leading causes of degenerative diseases. TAA is commonly used to induce acute or chronic liver disease by an increase a free radical thioacetamide-S-oxide and oxidative stress, resulting in necrosis of hepatocytes. We also demonstrated how the female reproductive organ, ovary, is affected by TAA treatment.

According to TAA treatment, liver cirrhosis was successfully induced, and it led to massive inflammation and accumulation of type 1 collagen and necrosis of hepatocytes. These changes were partially reverted by PD-MSC Tx. Regarding liver regeneration, the expression of hepatocyte regeneration factors, albumin and NOX4, were altered following TAA injection and PD-MSC Tx. According to previous studies, liver cirrhosis had been known as an irreversible liver disease. However, recent studies have discovered that stem cell therapy can be a cure for early stage cirrhosis and can replace liver transplant [15]. TAA injured model induced the trait of cirrhosis and those traits were ameliorated with PD-MSC Tx. Accumulation of collagen in TAA-injured rats liver models were reverted after PD-MSCs were transplanted in Sirius red, which suggested that PD-MSCs have antifibrotic effects. Transplantation of PD-MSCs also showed anti-inflammatory effect. Proinflammatory genes like IL-6 and TNF-α were upregulated when TAA chemical was treated and down-regulated when TAA chemical was treated and down-regulated when PD-MSCs were transplanted. In addition, PD-MSCs also appear to promote liver regeneration. Albumin and NOX4, essential factors that play an important role in maintaining hepatocyte structure, were down-regulated by TAA chemical and upregulated when PD-MSCs were transplanted. TAA induces liver cirrhosis by triggering oxidative stress which breaks the balance between reactive oxygen species (ROS) and antioxidant factors. In PD-MSCs transplanted model, antioxidant factors such as HO1, SOD1 and CAT showed decreased expression in TAA-injured rat model, and appeared increased expression in PD-MSCs transplanted model both *in vivo* and *in vitro* model which suggests that transplanting PD-MSCs harbor antioxidant effect.

One of the causes of reproductive diseases including endometriosis, polycystic ovary syndrome and unexplained infertility is oxidative stress induced from decreased antioxidant concentrations [16]. Excessive extracellular matrix (ECM) depositions following oxidative stress marked induced ovary dysfunction [17]. According to TAA treatment in ovary, accumulation of ECM in stromal cells nearby follicles is indicated in Masson Goldner trichrome staining and also based on decreased total number of follicles and increased number of atretic follicles (Figure 1). Previously, TAA treated ovary induced oxidative stress levels, resulting in imbalance of sex hormones [6]. ROS acts as an important signaling factor in granulosa cells, but also causes apoptosis when it exceeds normal level [18]. Since NOX4 level is restored to the normal level, we can conclude that the PD-MSCs treatment leads to normal ovarian function. Increase in HO1 expression in NTx group is supported by previous studies that HO1 is induced by ROS stimuli [19]. As an antioxidant defense stimulus, HO1 is known to suppress secretion of proinflammatory cytokines including IL-6, and it is supported by this study shown as Figure 2E [20]. As HO1 decreased in expression after PD-MSC Tx, the key factor of antioxidants SOD1 and CAT increased in expression. Previous reports suggested that SOD detoxifies SO anion to H_2_O_2_ and it is further degraded by CAT [16]. As a whole, when PD-MSCs is treated to ovary damaged with oxidative stress, NOX4 increases and activates granulosa cell signaling and proliferation, SOD1 and CAT increases and degrades ROS, and decreases HO1 which is induced by ROS. Therefore, restoration of ovarian function is supported by increase in folliculogenesis related genes and E2 hormone after PD-MSCs treatment.

**Figure 7:**
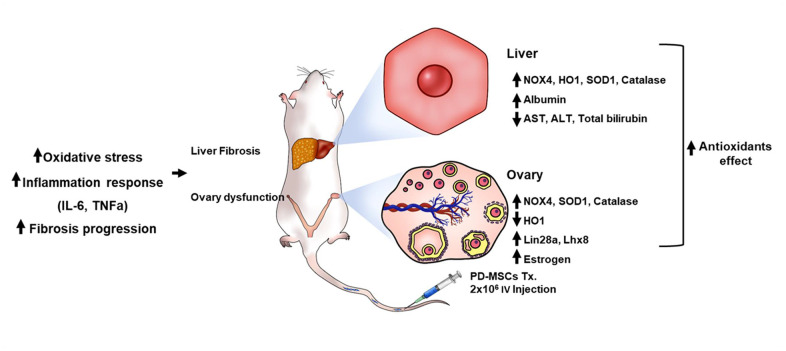
**The recovery pathway of TAA-injured rat model according to PD-MSCs transplantation.** The TAA-injured rat model lead to inflammatory response of serum, oxidative stress and fibrosis progression of liver and ovary in TAA-injured rat model. However, PD-MSCs transplantation decreased oxidative stress factors and increased antioxidants factors (e.g., SOD1 and Catalase) and functional restoration factors (e.g., Albumin and Lhx8) in liver and ovary of TAA-injured rat model. Also, functional restoration factors (e.g., Albumin, Lin28a, Lhx8 and E2) improved in liver and ovary of TAA-injured rat model. Therefore, PD-MSCs restore the liver and ovary function in TAA-injured rat model via antioxidants effect. These finding suggest new therapeutic strategy and support the fundamental mechanisms of stem cell therapy.

As shown in Figure 7, these findings show that TAA treatment damages both liver and ovary by triggering oxidative stress. PD-MSCs transplantation to this damaged model induces antioxidant activities which restores liver and ovary function by activating regeneration and folliculogenesis factors respectively. Although the hypothalamus-pituitary feedback pathway is the main signaling pathway of hormones related to ovarian function, reasonable conjectures about the relationship between liver and ovary could be made based on plausible evidence. The damage triggered in ovary can be explained by estrogen sulfotransferase (EST) in liver being damaged from TAA treatment, which then causes dysfunction in ovary [21]. Further studies should be done on the relationship between the liver and ovary caused by oxidative stress induced by TAA.

## MATERIALS AND METHODS

### Human placenta-derived mesenchymal stem cells culture

Human placenta samples were approved by the institutional review board of CHA general hospital, Seoul, Korea (IRB 07-18). PD-MSCs (38±2 gestational weeks) were isolated from the placenta and were cultured in 10% fetal bovine serum (FBS; Gibco), 1ug/ml Heparin (SIGMA-ALDRICH), 25ng/ml FGF4 (Peprotech), 1% Penicillin/streptomycin (Gibco) and MEM-alpha (HyClone) at 37° C under 5% CO2 incubator.

### TAA-injured rat model and transplantation of PD-MSCs.

All animal experiments were approved by the Institutional Animal Care and Use Committee (IACUC 190048) of the CHA laboratory animal research center in Korea. 7-week-old-female Sprague-Dawley rats (Orient Corporation) were maintained in an air-conditioned pathogen free animal facility at room temperature (21° C). Liver failure and ovary damage were induced by intraperitoneal (i.p.) injection of TAA (300mg/kg, SIGMA-Aldrich) twice a week for 12 weeks. PD-MSCs were stained using a PKH67 Fluorescent Cell Linker Kit (SIGMA-Aldrich) and transplanted via tail vein (2x10^6^ cells) after 8 weeks. The liver and ovaries were collected after 4 weeks of transplantation.

### Primary hepatocyte isolation

7-week-old Sprague Dawley rats (Orient Bio Inc., Seongnam, Korea) were maintained under specific pathogen-free conditions and were allowed to food before primary hepatocyte isolation. Hepatocytes were isolated using collagenase perfusion method [22] and seeded at a density of 3 x 10^5^ cells on 6-well plate. After 3 hrs, treatment with 70mM of TAA was performed, and then PD-MSCs (6 x 10^4^ cells) co-cultured with primary hepatocyte using 8-μm pore size Trans well insert. Hepatocytes were maintained in William’s E medium (Sigma-Aldrich, St. Louis, MO, US) supplemented with 10% FBS (Invitrogen), 1% Penicillin/streptomycin (P/S; Invitrogen), and 4 mM L-glutamine (Invitrogen). All animals were handled in accordance with the experimental protocols approved by the Institutional Animal Care Committee of CHA University, Seongnam Korea (IACUC-190048).

### Ovarian explant culture

In ovarian explant culture, ovary tissues isolated from the female SD rats were cut into half and placed on a 24-well with Matrigel (BD Bioscience) on the bottom and filled with media with FBS. Halved ovary was placed as the sliced portion facing up. After 70mM of TAA treatment for 24 hours, every wells went through a full media change and replaced the serum free media. Inserts of 8μm pore size (Falcon) containing PD-MSCs (1x10^4^ cells) were placed on top of every well for 24 hours. Harvested all samples for primary hepatocyte and ovary tissues were analyzed by qRT-PCR and Western blot analysis according to TAA treatment and PD-MSC co-cultivation.

### Quantitative RT-PCR

Total RNA was extracted from the liver and ovary samples using Trizol reagent (Invitrogen), chloroform (SIGMA-Aldrich) and isopropanol (Merck). RNA was reversely transcribed into cDNA using Superscript III and RNase out (Invitrogen). cDNA was amplified with designed primer and detected using a SYBR green master mix (ROCHE Diagnostics). The rat GAPDH was used for internal control. All experiments were performed in triplicate. Primer sequences for the ROS, antioxidant, liver regeneration and folliculogenesis factors are listed in Supplementary Table 1.

### Western blot analysis

All liver and ovary tissues were homogenized with LN2 and lysed on ice with RIPA buffer (SIGMA-Aldrich) containing protease inhibitor cocktail (Roche Diagnostics) and a phosphate inhibitor (A.G Scientific, Inc.). Protein lysates were separated by sodium dodecyl sulfate-polyacrylamide gel electrophoresis (SDS-PAGE), and transferred to polyvinylidene difluoride membranes (PVDF; Bio-Rad). The membrane is blocked with 5% BSA (Amresco) for 1 hour at RT, and then incubated with primary antibodies for overnight at 4° C. Antibodies were used in this study included rabbit anti-NOX4 (1:1000, Abcam), mouse anti-HO-1 (1:1000, Novus) rabbit anti-Catalase (1:1000, Abcam), mouse anti-SOD1 (1:1000, Cell Signaling), rabbit anti-albumin (1:500, Novus), mouse anti-Nobox (1:500, Santa Cruz), rabbit anti-Nanos3 (1:1000, Abcam), goat anti-Lhx8 (1:500, Santa Cruz), rabbit anti-GAPDH (1:1000, Abfrontier) and horseradish peroxidase – (HRP-) conjugated secondary antibody (anti-rabbit IgG; 1:10000; Cell signaling and anti-mouse IgG antibody; 1:5000, Cell Signaling). All experiments were performed in triplicate. Intensity of each band was quantified by Image J software (NIH, Bethesda, http://www.nih.gov).

### Enzyme-linked immunosorbent assay (ELISA)

To analyze the inflammatory reaction in TAA-injured rat model, IL-6 and TNF-alpha concentration in serum of animal were measured using by a Millipore rat metabolic magnetic bead panel by Yujung Meditec company. IL-10 concentration in serum was measured using by Elisa assay kit (R&D). The serum concentration of total aspartate transaminase (AST), alanine transaminase (ALT), bilirubin and albumin were measured for analyze the liver function by Southeast Medi-Chem Institute (SEMI). To analyze the ovary function, enzyme-linked immunosorbent assay (ELISA) kit for estradiol (E2) (EKU03971, Biomatik) was used to measure the level of E2 in the supernatant of the media from the explant cultured ovaries. The procedures were performed according to the manufacturer’s protocol. All experiments were performed in triplicate.

### Histological analysis

Liver and ovary tissue samples were sampled in 10% formalin, ingrained in paraffin and sliced at 4 μm thickness. Then, Hematoxylin & Eosin (H&E), Sirius Red and Masson Goldner staining is performed on the sections (T&P Bio company) and observed under Panoramic digital slide scanners (3D HISTECH Ltd.) at 200x magnification. All stained tissues were analyzed and measured by Cell Quant and Histo Quant software (3D HISTECH Ltd.).

### PKH67 labeled PD-MSCs detection

To analyze the engraftment of PD-MSCs in the liver and ovary tissues, 7 μm thick cryostat sections were washed with phosphate-buffered saline (PBS; Elbio), and then the sections were dyed with 4’,6-diamidino-2-phenylindole (DAPI) for nuclear staining and observed under fluorescence microscopy at 400x magnification (ZEISS).

### Mito SOX and Mito Tracker staining

To analyze the superoxide accumulation, sectioned tissues were washed with PBS, and then sectioned tissue were stained with Mito SOX (50nM/ml; Invitrogen) and Mito Tracker (500mM/ml; Invitrogen) solution with PBS for superoxide staining in tissue and observed under fluorescence microscope at x100 and x400 magnification (Zeiss).

### Statistical analysis

All experiments were conducted in duplicate or triplicate manner. Data that are used in the graph or chart is expressed as mean ± standard deviation. Student’s t-test was performed to prove statistical significance among the groups and *p*<0.05 was considered statistically significant. Statistical analyses were accomplished using PASW version 22.0 (SPSS Inc., Chicago, IL, USA).

## Supplementary Material

Supplementary Figures

Supplementary Tables

## References

[1] Pizzino G, Irrera N, Cucinotta M, Pallio G, Mannino F, Arcoraci V, Squadrito F, Altavilla D, Bitto A. Oxidative stress: harms and benefits for human health. Oxid Med Cell Longev. 2017; 2017:8416763. 10.1155/2017/8416763 28819546 PMC5551541

[2] Mead B, Berry M, Logan A, Scott RA, Leadbeater W, Scheven BA. Stem cell treatment of degenerative eye disease. Stem Cell Res. 2015; 14:243–57. 10.1016/j.scr.2015.02.003 25752437 PMC4434205

[3] Shi Y, Wang Y, Li Q, Liu K, Hou J, Shao C, Wang Y. Immunoregulatory mechanisms of mesenchymal stem and stromal cells in inflammatory diseases. Nat Rev Nephrol. 2018; 14:493–507. 10.1038/s41581-018-0023-5 29895977

[4] Jung J, Choi JH, Lee Y, Park JW, Oh IH, Hwang SG, Kim KS, Kim GJ. Human placenta-derived mesenchymal stem cells promote hepatic regeneration in CCl4-injured rat liver model via increased autophagic mechanism. Stem Cells. 2013; 31:1584–96. 10.1002/stem.1396 23592412

[5] Wallace MC, Hamesch K, Lunova M, Kim Y, Weiskirchen R, Strnad P, Friedman SL. Standard operating procedures in experimental liver research: thioacetamide model in mice and rats. Lab Anim. 2015 (Suppl 1); 49:21–29. 10.1177/0023677215573040 25835735

[6] Lee YH, Son JY, Kim KS, Park YJ, Kim HR, Park JH, Kim KB, Lee KY, Kang KW, Kim IS, Kacew S, Lee BM, Kim HS. Estrogen deficiency potentiates thioacetamide-induced hepatic fibrosis in sprague-dawley rats. Int J Mol Sci. 2019; 20:3709. 10.3390/ijms20153709 31362375 PMC6696236

[7] Chilakapati J, Shankar K, Korrapati MC, Hill RA, Mehendale HM. Saturation toxicokinetics of thioacetamide: role in initiation of liver injury. Drug Metab Dispos. 2005; 33:1877–85. 10.1124/dmd.105.005520 16183780

[8] Low TY, Leow CK, Salto-Tellez M, Chung MC. A proteomic analysis of thioacetamide-induced hepatotoxicity and cirrhosis in rat livers. Proteomics. 2004; 4:3960–74. 10.1002/pmic.200400852 15526343

[9] Wang H, Ruan X, Li Y, Cheng J, Mueck AO. Oxidative stress indicators in Chinese women with PCOS and correlation with features of metabolic syndrome and dependency on lipid patterns. Arch Gynecol Obstet. 2019; 300:1413–21. 10.1007/s00404-019-05305-7 31549221

[10] Pop DM, SoriŢău O, Şuşman S, Rus-Ciucă D, Groza IŞ, Ciortea R, Mihu D, Mihu CM. Potential of placental-derived human mesenchymal stem cells for osteogenesis and neurogenesis. Rom J Morphol Embryol. 2015; 56:989–96. 26662130

[11] Komaki M, Numata Y, Morioka C, Honda I, Tooi M, Yokoyama N, Ayame H, Iwasaki K, Taki A, Oshima N, Morita I. Exosomes of human placenta-derived mesenchymal stem cells stimulate angiogenesis. Stem Cell Res Ther. 2017; 8:219. 10.1186/s13287-017-0660-9 28974256 PMC5627451

[12] Ávila J, González-Fernández R, Rotoli D, Hernández J, Palumbo A. Oxidative stress in granulosa-lutein cells from *in vitro* fertilization patients. Reprod Sci. 2016; 23:1656–61. 10.1177/1933719116674077 27821562

[13] Crosas-Molist E, Bertran E, Rodriguez-Hernandez I, Herraiz C, Cantelli G, Fabra À, Sanz-Moreno V, Fabregat I. The NADPH oxidase NOX4 represses epithelial to amoeboid transition and efficient tumour dissemination. Oncogene. 2017; 36:3002–14. 10.1038/onc.2016.454 27941881 PMC5354266

[14] Basmaeil YS, Al Subayyil AM, Khatlani T, Bahattab E, Al-Alwan M, Abomaray FM, Kalionis B, Alshabibi MA, AlAskar AS, Abumaree MH. Human chorionic villous mesenchymal stem/stromal cells protect endothelial cells from injury induced by high level of glucose. Stem Cell Res Ther. 2018; 9:238. 10.1186/s13287-018-0984-0 30241570 PMC6150972

[15] Yin F, Wang WY, Jiang WH. Human umbilical cord mesenchymal stem cells ameliorate liver fibrosis *in vitro* and *in vivo*: from biological characteristics to therapeutic mechanisms. World J Stem Cells. 2019; 11:548–64. 10.4252/wjsc.v11.i8.548 31523373 PMC6716089

[16] Agarwal A, Aponte-Mellado A, Premkumar BJ, Shaman A, Gupta S. The effects of oxidative stress on female reproduction: a review. Reprod Biol Endocrinol. 2012; 10:49. 10.1186/1477-7827-10-49 22748101 PMC3527168

[17] Takahashi N, Harada M, Hirota Y, Nose E, Azhary JM, Koike H, Kunitomi C, Yoshino O, Izumi G, Hirata T, Koga K, Wada-Hiraike O, Chang RJ, et al. Activation of endoplasmic reticulum stress in granulosa cells from patients with polycystic ovary syndrome contributes to ovarian fibrosis. Sci Rep. 2017; 7:10824. 10.1038/s41598-017-11252-7 28883502 PMC5589802

[18] Yang H, Xie Y, Yang D, Ren D. Oxidative stress-induced apoptosis in granulosa cells involves JNK, p53 and Puma. Oncotarget. 2017; 8:25310–22. 10.18632/oncotarget.15813 28445976 PMC5421932

[19] Tron K, Samoylenko A, Musikowski G, Kobe F, Immenschuh S, Schaper F, Ramadori G, Kietzmann T. Regulation of rat heme oxygenase-1 expression by interleukin-6 via the Jak/STAT pathway in hepatocytes. J Hepatol. 2006; 45:72–80. 10.1016/j.jhep.2005.12.019 16510205

[20] Jiao Y, Ye DZ, Li Z, Teta-Bissett M, Peng Y, Taub R, Greenbaum LE, Kaestner KH. Protein tyrosine phosphatase of liver regeneration-1 is required for normal timing of cell cycle progression during liver regeneration. Am J Physiol Gastrointest Liver Physiol. 2015; 308:G85–91. 10.1152/ajpgi.00084.2014 25377314 PMC4380483

[21] Cooke PS, Nanjappa MK, Ko C, Prins GS, Hess RA. Estrogens in male physiology. Physiol Rev. 2017; 97:995–1043. 10.1152/physrev.00018.2016 28539434 PMC6151497

[22] Stoeckman AK, Towle HC. The role of SREBP-1c in nutritional regulation of lipogenic enzyme gene expression. J Biol Chem. 2002; 277:27029–35. 10.1074/jbc.M202638200 12016216

