# Supplementary material for: Human placenta-derived mesenchymal stem cells trigger repair system in TAA-injured rat model via antioxidant effect
Source: Aging (Albany NY). 2020 Dec 26;13(1):61–76. doi: 10.18632/aging.202348 (PMC7835021; doi:10.18632/aging.202348)
Supplement: Supplementary Figures [file aging-13-202348-s001.pdf]

SUPPLEMENTARY FIGURES

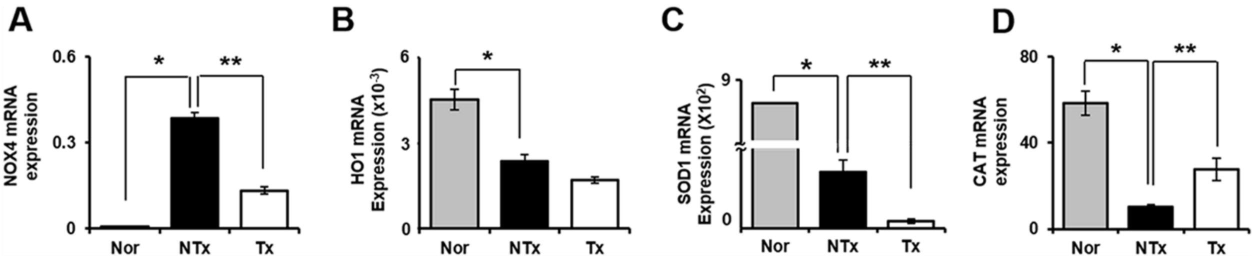

Supplementary Figure 1. Antioxidants effect of PD-MSCs in TAA-injured rat liver. The mRNA expression related to antioxidants factors were analyzed in TAA-injured rat liver according to PD-MSCs co-cultivation by qRT PCR (**A**–**D**). Data represent the mean ± S.D. * Significantly different versus Normal (*$p<0.05$). ** Significantly different versus NTx (**$p<0.05$).

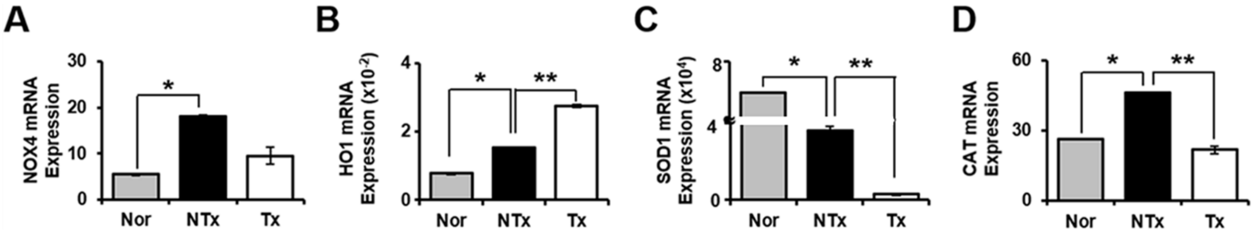

Supplementary Figure 2. Antioxidants effect of PD-MSCs in TAA-injured rat ovary. The mRNA expression related to antioxidants factors (**A**–**D**) were analyzed in TAA-injured rat ovary according to PD-MSCs co-cultivation by qRT-PCR. Data represent the mean ± S.D. * Significantly different versus Normal (*$p<0.05$). ** Significantly different versus NTx (**$p<0.05$).

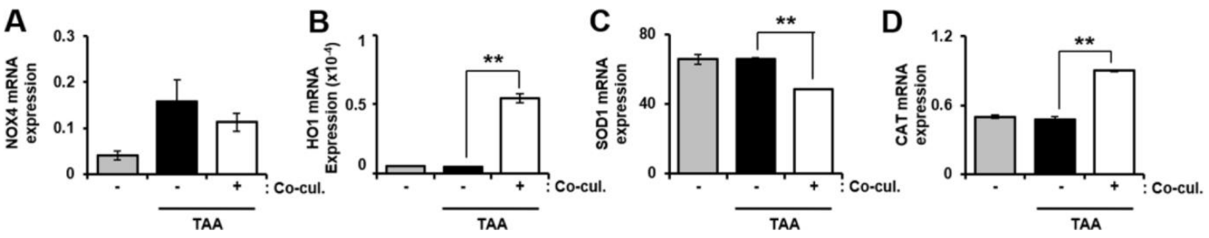

Supplementary Figure 3. Antioxidants effect of PD-MSCs in TAA-treated rat hepatocytes. The mRNA expression related to antioxidants factors were analyzed in TAA-treated rat hepatocytes according to PD-MSCs co-cultivation by qRT-PCR (**A**–**D**). Data represent the mean ± S.D. * Significantly different versus Normal (*$p<0.05$). ** Significantly different versus NTx (**$p<0.05$).

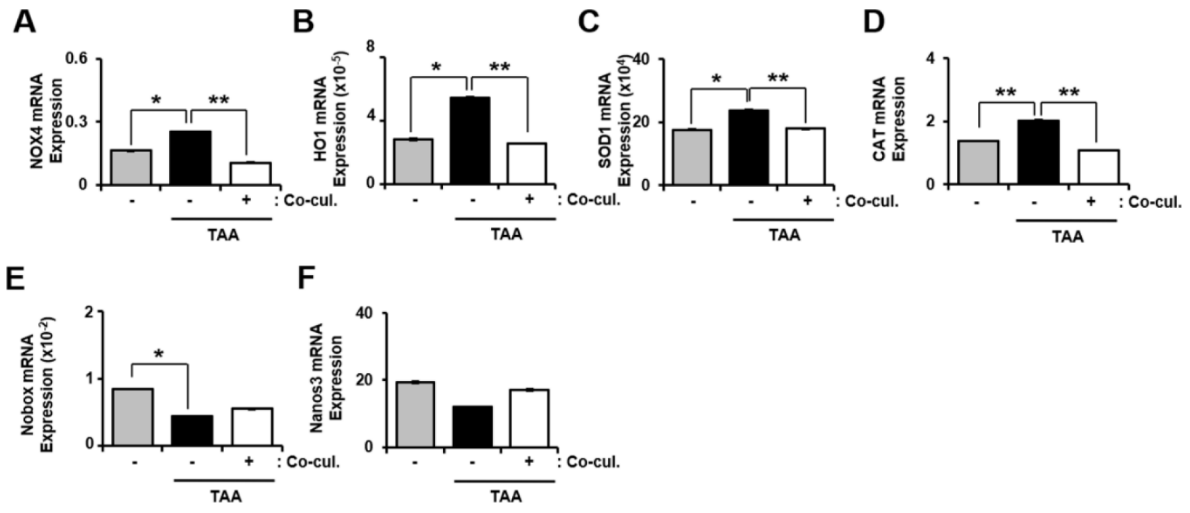

Supplementary Figure 4. Antioxidants effect of PD-MSCs in TAA-treated ovary in *ex vivo*. The mRNA expression related to antioxidants factors and (**E**, **F**) folliculogenesis were analyzed in ovary of TAA-treated ovary according to PD-MSCs co-cultivation by qRT-PCR (**A**–**D**). Data represent the mean ± S.D. * Significantly different versus Normal (*$p<0.05$). ** Significantly different versus NTx (**$p<0.05$).
