# Supplementary material for: Human placenta-derived mesenchymal stem cells trigger repair system in TAA-injured rat model via antioxidant effect
Source: Aging (Albany NY). 2020 Dec 26;13(1):61–76. doi: 10.18632/aging.202348 (PMC7835021; doi:10.18632/aging.202348)
Supplement: Supplementary Tables [file aging-13-202348-s002.pdf]

SUPPLEMENTARY TABLES

Supplementary Table 1. Primer sequences used in quantitative real time polymerase chain reaction.

Gene	Accession Number	Sequence (5' – 3')	Tm (℃)
NOX4	NM_053524.1	F: 5'-AGGTGTCTGCATGGTGGTG-3'<br>R: 5'-GAGGGTGAGTGTCTAAATTGGT-3'	55
HO1	NM_012580.2	F: 5'-TGCACATCCGTGCAGAGAAT-3'<br>R: 5'-CTGGGTTCTGCTTGTTTCGC-3'	55
SOD	NM_017050.1	F: 5'-TTTTGCTCTCCCAGGTTCCG-3'<br>R: 5'-TGTCCTGACACCACAACTGG-3'	55
CAT	NM_012520.2	F: 5'-ACGAGATGGCACACTTTGACAG-3'<br>R: 5'-ACACCGGGGACCAAATGATG-3'	55
ALB	NM_134326.2	F: 5'-CTTCAAGCCTGGGCAGTAG-3'<br>R: 5'-GCACTGGCTTATCACAGCAA-3'	60
Lhx8	NM_001012219.1	F: 5'-GTATCACTTGGCTTGCTT-3'<br>R: 5'-ATTACCGTTCTCCACTTC-3'	55
Nanos3	NM_001105945.1	F: 5'-CTCTGCATGAGGAAGAGGAGC-3'<br>R: 5'-GGACTGATAGATCGCACGAG-3'	55
Nobox	NM_001192013.1	F: 5'-AGCCAGTGCAGATCTGCACC-3'<br>R: 5'-TGTCACTGCCAGGAACATCCCTC-3'	55
Lin28a	NM_001109269.1	F: 5'-CCCGGTGGACGTCTTTGTG-3'<br>R: 5'-CACTGCCTCACCCTCCTTGA-3'	55
GAPDH	NM_017008.4	F: 5'-TCCCTCAAGATTGTCAGCAA-3'<br>R: 5'-AGATCCACAACGGATACATT-3'	60

Supplementary Table 2. Biochemical analysis of serum parameters in TAA-injured rat model.

Group	AST (U/L)	ALT (U/L)	Total bilirubin (μmol/L)	Albumin (g/L)
Normal (n=6)	85.06 ± 6.94	43.63 ± 2.14	0.86 ± 0.22	43.11 ± 0.97
NTx (n=7)	168.06 ± 12.52*	117.32 ± 5.97*	11.275 ± 0.42*	31.09 ± 1.17*
Tx (n=6)	124.48 ± 5.05*, **	86.73 ± 4.07*, **	6.32 ± 0.74*, **	37.42 ± 0.47*, **

ALT, Alanine aminotransferase; AST, Aspartate transaminase. * Significantly different versus Normal (*$p<0.05$). ** Significantly different versus NTx (** $p<0.05$).
